# Impact of Soy Protein Concentrate and Storage on the Safety, Quality and Shelf Stability of Beef Patties

**DOI:** 10.1002/fsn3.71327

**Published:** 2025-12-26

**Authors:** Muhammad Moazzam, Sher Ali, Muhammad Ammar Khan, Muhammad Waseem, Muhammad Rizwan Javed, Nasir Rajput, Tawfiq Alsulami, Kashif Nauman, Crossby Osei Tutu

**Affiliations:** ^1^ Department of Meat Science and Technology, Faculty of Animal Production and Technology University of Veterinary and Animal Sciences Lahore Pakistan; ^2^ Institute of Food Science and Technology Sindh Agriculture University Hyderabad Pakistan; ^3^ Department of Food Science and Technology, Faculty of Agriculture and Environment Islamia University of Bahawalpur Bahawalpur Pakistan; ^4^ College of Ocean Food and Biological Engineering Jimei University Xiamen China; ^5^ Department of Poultry Husbandry, Faculty of Animal Husbandry and Veterinary Sciences Sindh Agriculture University Tandojam Tandojam Pakistan; ^6^ Department of Food Science and Nutrition, College of Food and Agricultural Sciences King Saud University Riyadh Saudi Arabia; ^7^ Department of Family and Consumer Sciences University of Ghana, Legon Accra Ghana

**Keywords:** chilling storage, meat, proteins, ready to eat, soy, value addition

## Abstract

Incorporating affordable, plant‐based alternatives into beef products presents challenges such as compromised quality, nutritional value, and consumer acceptance. This study evaluated the effects of rehydrated soy protein concentrate (SPC; 1:2 SPC‐to‐water ratio) at 0%, 10%, 15%, 20%, and 25% inclusion levels on the physicochemical, microbiological, textural, and sensory properties of beef patties. Patties were stored at 2°C ± 2°C for 7 days to assess stability. At SPC_25%_, beef patties showed significantly higher pH (5.96), water‐holding capacity (80.70%), cooking yield (83.07%), and reduced diameter shrinkage (19.02%). Color analysis revealed higher lightness (L* = 45.52), yellowness (b* = 11.91), and lower redness (a* = 12.36). Thiobarbituric acid reactive substances (TBARS) values were lower (*p* < 0.05), while microbial counts were higher (*p* < 0.05) in SPC patties on day 7. Sensory evaluation showed improved mouthfeel, juiciness, and tenderness, particularly in SPC_25%_ patties. Texture profile analysis confirmed reduced hardness (25.2 N) and gumminess (15.4 N). Overall, SPC‐enhanced patties offered better water retention, texture, and yield. These improvements, alongside extended oxidative stability, suggest SPC suitability as a functional meat replacer in products targeted at older or health‐conscious consumers.

## Graphical abstract

Soy protein concentrate (SPC) at 25% increased WHC and decreased shrinkage in patties. SPC enhanced shelf life by halting microbial growth owing to bioactive compounds. The yellow line represents the decreasing trend of redness (a*), while the blue line shows the increasing trends of lightness (L*) and yellowness (b*).

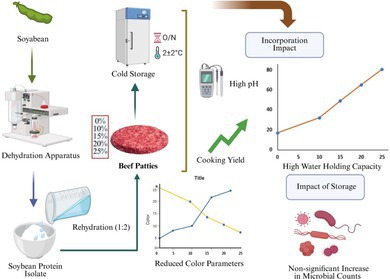

## 1 Introduction

Beef is a staple diet in all regions of the globe (Murimi 2022; Suleman et al. 2025), owing to its nutrient‐dense attributes and its high biological value protein, iron, essential fatty acids (Murimi 2022; Suleman et al. 2025), B_12_, B_6_ vitamins, zinc, and phosphorus (Egelandsdal et al. 2020), proteinogenic amino acids, taurine and creatine (Wu 2021). An intense beef flavor is a primary reason for consumers preferring beef over chicken (Kerth and Miller 2015; Adil et al. 2025b; Javed et al. 2025). Beef mince often has a short shelf life primarily due to oxidative damage and higher microbial spoilage (Mitsumoto et al. 2005; Adil et al. 2025a). However, the role of beef in the human diet, due to its proteins, lipids, trace elements, and vitamins is crucial for development and sustainability (Pighin et al. 2016; Williams and Hill 2017; Acheampong et al. 2025a). Amino acids obtained from food of animal origin have superior quality, better digestibility and higher functionality (Wolk 2017; Waseem et al. 2024; Javed et al. 2025; Osei Tutu et al. 2024b). Excessive dietary intake of beef (> 70.0 g day; National Health Service, UK) may result in health risks such as colorectal adenoma, lung cancer, coronary heart disease, and stroke (Grosso et al. 2022; Acheampong et al. 2025b), esophageal, and gastric malignancies (Lippi et al. 2016), which could be due to systemic oxidative stress, free radicals formation, and inflammation (van Hecke et al. 2016). Moreover, livestock production can have major negative effects on the environment (Godfray et al. 2018; Khan et al. 2025; Acheampong et al. 2025). Growing population and industrialization have led to massive demand for animal proteins (Laestadius et al. 2013; Steinfeld et al. 2006; Wang et al. 2023). Furthermore, sustainable development goals (SDG) # 2, # 3, and # 12 commit that consumers get safe, sustainable, nutritious, and affordable food (Pintado and Delgado Pando 2020; Asiedu et al. 2026; Kumador et al. 2025; Mahama et al. 2025a). It helps in improving health, growth and development (Leroy et al. 2023; Osei Tutu et al. 2024a; Mahama et al. 2025b). Blending animal‐derived proteins with plant‐based proteins may be a solution (Wu et al. 2013; Amjad et al. 2026).

In meat processing the nutritional and eating quality is improved owing to the inclusion of healthy plant ingredients and the removal of unhealthy ingredients like cholesterol, salt, additives, and unsaturated fatty acids (Jiménez‐Colmenero et al. 2001; Pintado and Delgado Pando 2020; Nyasordzi et al. 2025). A study by (Wang et al. 2023) reported the use of several strategies including proteins from plant origin, and cultured meat owing to their more environmentally friendly, economical, healthier and lower carbon footprints (Fresán et al. 2019; Tilman and Clark 2014), therefore the research has increased recently in this direction (Aiking 2011; Lee et al. 2021; Akonor et al. 2023c). Plant‐based proteins, such as soy, wheat and corn, aid in binding, extending, filling, and emulsification properties in meat systems (Hong et al. 2022). Using plant powders in beef patties can improve oxidative stability (Duthie et al. 2013; Osei Tutu et al. 2023; Akonor et al. 2023b), increase dietary fibers, reduce microbes (Bermúdez et al. 2023), enhance nutrients, and reduce costs (Chaudhary and Tremorin 2020), though it may affect texture and sensory qualities (Bermúdez et al. 2023; Akonor et al. 2023a). Moreover, they increase the structural integrity and functionality of meat products (Shen et al. 2022). Consumer acceptance of plant‐based additives in meat dishes has paved a path to develop healthier and sustainable meat (Pintado and Delgado Pando 2020).

Plant‐derived proteins, often serving as meat extenders, stand out for being high‐value protein sources at a low cost with good nutrition (Kortei et al. 2024; Ozmen et al. 2025; Haider et al. 2025a). Fibers and proteins in meat enhance cooking yield, taste and nutrients (Petracci et al. 2013; Shen et al. 2022). Akesowan (2010) demonstrated that integrating 2% soy protein isolate (SPC) in pork burgers led to better texture e.g., cohesiveness, springiness and chewiness. Similarly, Hidayat et al. (2018) also reported that a 30% substitution of texturized vegetable proteins into beef sausages improved cooking yield, sensory attributes and reduced hardness. Also, Yi et al. (2012) added rice flour in beef patties and exhibited reduced cooking losses. Therefore, it is prudent to explore SPC as a cost‐effective meat replacer in beef patties with better nutrition, texture and quality (Bernasconi et al. 2020). SPC halts lipid oxidation and pigment discoloration (Das, Anjaneyulu, Verma, and Kondaiah 2008). Soy ingredients like proteins, flour, isolates, defatted flour/flakes, and SPC have appeared in both vegan and lactose‐free products (Kumar et al. 2022; Lyu et al. 2022; Preece et al. 2017), meatballs, sausages, and burgers which enhance emulsifying and gelling properties, cost‐effectiveness, and superior product texture (Balestra and Petracci 2019), and reduced health risks because of isoflavones, saponin and lecithin (Mistry et al. 2020; Muramatsu et al. 2017), dietary fiber, and digestibility (Sipos 2013).

Despite growing interest in plant‐based protein alternatives, limited research has utilized SPC for maintaining the quality of beef at storage. Therefore, the present study aimed to enhance the applicability of SPC for improving shelf stability, optimizing SPC‐fortified beef patties for extended life and quality attributes for commercial viability, and higher consumer acceptability.

## 2 Material and Methods

### 2.1 Procurement of Raw Materials

#### 2.1.1 Experimental Design and Sampling

The visually lean meat of beef cattle (carcass weight 140 ± 10 kg, aged 18–20 months) was procured from the commercial slaughter house and sales center Meat Science & Technology Department, University of Veterinary and Animal Sciences, Lahore (Pakistan). The total minced meat required for forming total patties was ≃18 kg. Visually lean beef with 15% added fat was passed through a mincer with an 8 mm aperture size plate (C‐E800, Minerva Omega, Italy). SPC (protein 65%–70% commercially, with the remaining ingredients as inert components e.g., carbohydrates, residual moisture and fibers) was purchased from a local vendor and hydrated for 1 h (1:2, SPC: chilled distilled water). Thereafter, 1.5% salt, 0.25% black pepper, and 0%–25% SPC were added and mixed well using a mixer (Omega Food Tech, Italy). Settling was done at 2°C ± 2°C for 24 h before cooking and performing experiments. Three batches for each treatment were prepared (after each week). Samples for all 5 treatment levels were prepared in 3 batches and tested accordingly, weighing 100 ± 2 g each (100 mm diameter and 12 mm thickness) were formed using a manual burger maker (Model AM13, Hebei, China). All the patties were placed in thermocol disposable trays, wrapped with oxygen‐permeable film, and stored in the display chiller (S80100, Tecnodom S.P.A, Italy) at 2°C ± 2°C for 7 days and all the following quality and safety assessments were performed on the 1st, 3rd, 5th, and 7th days of the refrigerated storage.

### 2.2 Shelf Stability

#### 2.2.1 pH Values Determination

A pH meter (ProfiLine, WTW, pH 3110 SET 2, Germany), equipped with a penetration glass probe calibrated at pH 4 and 7, was implemented to measure the pH values of beef patty samples three times at three different points in each patty on each study day.

#### 2.2.2 Estimation of Water Activity (a_w_)

The water activity (a_w_) of samples was evaluated using a water activity meter (Lab‐Swift CH 8853, Switzerland) following the manufacturer's directions (Sharima‐Abdullah et al. 2018).

#### 2.2.3 Determination of Lipid Oxidation Measured as TBARS

A method of Ball et al. (2021) was used to determine the TBARS in beef patty samples. Accurately, in a water bath (WNB‐29, Memmert, Schwabach, Germany) set to 100°C, a solution comprising 2.5 mL of 0.375% TBA, 0.25 N HCl, and 15% trichloroacetic acid was heated for 10 min. Meanwhile, there were 2.0 g of patties to use for the sample. After heating, the sample was spun in a centrifuge (Hermle Z236K, Wehingen, Germany) at 5500 rpm for 25 min. From each test tube, the upper layer (supernatant) was separated, and the absorbance was taken at 532 nm while comparing with the reagent blank (distilled water) employing a UV‐160 spectrophotometer (Shimadzu UV‐1800, Kyoto, Japan). Following the malonaldehyde standard curve (0 to 2 ppm), the TBARS values were found and expressed as ppm using the following formula:

(1) $$\mathrm{TBA}\;\text{number}\;\left(\mathrm{ppm}\right)=\text{Absorbance of sample}\;\mathrm{at}\;532\;\mathrm{nm}\times 2.77$$

#### 2.2.4 Total Viable Count Measurement

Bacterial growth in the samples was determined following the method prescribed by Liang et al. (2022). A 10 g sample of the patty was placed in a stomacher bag containing 90 mL sterile 0.1% peptone water. The stomacher (Interscience, BagMixer 400P, France) homogenized the sample at 4 strokes per second for 2 min. After that, a 10‐fold serial dilution was prepared, and 0.1 mL samples from the dilutions prepared were spread on the nutrient agar plate. After incubation at 37°C for 48 h (duplicate), the microbial total viable count was done using the Quebec colony counter (WTW, BZG‐28, Weilheim, Germany), and the results are expressed as log_10_ CFU/g.

### 2.3 Assessment for Commercial Suitability

#### 2.3.1 Moisture Loss, Cooking Loss and Yield

The samples were fried in oil on the griddle attached to the oven (SY‐MS220SN, Guangzhou, Guangdong, China) at 176°C, until the core reached 72°C according to the thermometer (TP101, Fujian, China) with a measuring range from −50°C to 300°C. The cooking loss (%) was measured by using the following formula:

(2) $$\begin{array}{c} \text{Cooking loss}\;\left(\%\right)\\ =\frac{\text{Weight of}\;\mathrm{raw}\;\text{patty}-\text{Weight of cooked patty}}{\text{Weight of}\;\mathrm{raw}\;\text{patty}}\times 100 \end{array}$$

After cooking, the patties were allowed to cool to room temperature (30°C ± 2°C for 5 min) and the cooking yield was recorded using the following formula:

(3) $$\text{Cooking yield}\;\left(\%\right)=\frac{\text{Weight of cooked patty}}{\text{Weight of}\;\mathrm{raw}\;\text{patty}}\times 100$$

Each patty was weighed on a weighing balance (SF‐400, 7000 g × 1 g capacity, China).

The moisture loss (%) was determined using the following formula:

(4) $$\text{Moisture loss}\;\left(\%\right)=\frac{\text{Initial weight}-\text{Final weight}}{\text{Initial weight}}\times 100$$

#### 2.3.2 Diameter Shrinkage (%)

The diameter shrinkage (%) was determined as described by Eshag Osman et al. (2021) at three different points (triplicate measurements) before and after cooking, using the following formula:

(5) $$\begin{array}{c} \text{Diameter shrinkage}\;\left(\%\right)\\ =\frac{\text{Diameter of}\;\mathrm{raw}\;\text{patty}-\text{Diameter of cooked patty}}{\text{Diameter of}\;\mathrm{raw}\;\text{patty}}\times 100 \end{array}$$

#### 2.3.3 Expressible Moisture and Water‐Holding Capacity

The expressible moisture (%) was determined using the filter paper (Whatman No. 40) press technique to determine expressible juice percentage (Daniel et al. 2011; Haider et al. 2025c), using a compression machine (YYW‐2, Nanjing Soil Instrument, Nanjing, China) for 5 min with a force of 373 N, by the following formula:

(6) $$\text{Expressible moisture}\;\left(\%\right)=\frac{\text{Initial weight}-\text{Final weight}\;}{\text{Initial weight}}\times 100$$

The water‐holding capacity (WHC, %) was calculated from expressible moisture (%) using the following formula:

(7) $$\mathrm{WHC}\;\left(\%\right)\;100–\text{Expressible moisture}\;\left(\%\right)$$

### 2.4 Consumer Acceptability

#### 2.4.1 Color Evaluation

The CIE L* a* and b* color coordinates of cooked beef patties were thrice measured using a chromameter (Konica Minolta CR‐410, Tokyo, Japan), provided with D65 illuminant, 2° standard observer, and 50 mm aperture, calibrated with a white tile (Y = 85.40, x = 0.3178, y = 0.3338) (Ball et al. 2021; Haider et al. 2025b; Asiedu et al. 2025).

#### 2.4.2 Textural Profile Analysis

The analysis of the samples' textures was done at room temperature on 1 cm pieces using a texture profile analyzer (TA. XT plus texture analyzer manufactured by stable microsystem UK) with a cylindrical probe (SMP P/35, flat bottom, diameter 35 mm). Two tests were done at 50% of the original height with a pretest speed of 1.00 mm/s, test speed of 5.00 mm/s, post‐test speed of 5.00 mm/s, and a trigger force that was 20 g (Noor Hidayati et al. 2021; Mensah et al. 2026).

#### 2.4.3 Sensory Analysis

The sensory properties of the beef patties were judged by semi‐trained panelists (*n* = 20) consisting of faculty members and postgraduate students of the Department of Meat Science and Technology, UVAS, according to the American Meat Science Association Sensory Guidelines (AMSA 1995) as described by Hernandez et al. (2023). The panelists evaluated the properties including odor, juiciness, taste, tenderness, mouthfeel, and overall acceptability on a 9‐Point Hedonic Scale, with 1 being extremely disliked and 9 being extremely liked. Sensory evaluation was conducted in a controlled environment under ample white fluorescent lighting to ensure uniform visibility of samples, a noise‐free environment ensuring participant safety, with unrestricted access to clean drinking water, adequate lighting, and conditions designed to minimize bias throughout the assessment period.

### 2.5 Statistical Analysis

All experiments were done three times (i.e., *n* = 3), and the average results with their values were reported as mean ± S.D. The results from the analyses were studied using analysis of variance (ANOVA) on Statistics 8.1 (Tallahassee, FL, USA). Subsequently, the data were checked using the LSD test at a significance level of *p* < 0.05.

## 3 Results and Discussion

### 3.1 Shelf Stability

#### 3.1.1 pH Values

SPC supplementation in beef patties and storage for 0–7 days resulted in a non‐significant change in pH values from 5.75 to 5.81 and 5.75–5.95 for SPC_10%_–SPC_25%_ (10%–25%) at storage interval (0–7 days), respectively (Table 1). Our findings align with Kamani et al. (2019) study, in which the addition of plant proteins in chicken meat sausages increased the pH values. Similarly, Ahmad et al. (2010) reported that the addition of SPC in buffalo meat sausages significantly (*p* < 0.05) increased pH values. In contrast to Hidayat et al. (2018), the results reported that the substitution of beef sausages with soy‐based texturized vegetable protein and inclusion of soy protein in goat meat nuggets did not significantly affect the pH values. On the other hand, there was no effect of storage days on the pH of SPC‐added raw beef patties. The results of this study were in contrast to the findings of Ahmad et al. (2010) reported a decrease in pH in beef emulsion sausages having 0%–25% SPC during the storage period of 28 days.

**TABLE 1:** Effect of soy protein concentrate (SPC) supplementation on pH of beef patties stored under refrigeration at 1–7 days.

Days	SPC_0%_	SPC_10%_	SPC_15%_	SPC_20%_	SPC_25%_
1	5.75 ± 0.13^Ab^	5.94 ± 0.06^Aa^	5.95 ± 0.03^Aa^	5.95 ± 0.03^Aa^	5.95 ± 0.08^Aa^
3	5.76 ± 0.13^Ab^	5.95 ± 0.05^Aa^	5.94 ± 0.02^Aa^	5.94 ± 0.03^Aa^	5.96 ± 0.08^Aa^
5	5.79 ± 0.13^Ab^	5.94 ± 0.06^Aa^	5.94 ± 0.03^Aa^	5.95 ± 0.04^Aa^	5.96 ± 0.08^Aa^
7	5.81 ± 0.13^Ab^	5.93 ± 0.07^Aa^	5.92 ± 0.03^Aa^	5.93 ± 0.04^Aa^	5.95 ± 0.08^Aa^

*Note:* Capital lettering exhibiting statistically significant (*p* < 0.05) mean values in a column, while; small lettering exhibiting statistically significant (*p* < 0.05) mean values in a row. SPC_0%_ = 0% soy protein concentrate supplemented beef patties (Control), SPC_10%_ = 10% soy protein concentrate supplemented beef patties, SPC_15%_ = 15% soy protein concentrate supplemented beef patties, SPC_20%_ = 20% soy protein concentrate supplemented beef patties, SPC_25%_ = 25% soy protein concentrate supplemented beef patties.

#### 3.1.2 Water Activity

**FIGURE 1:**
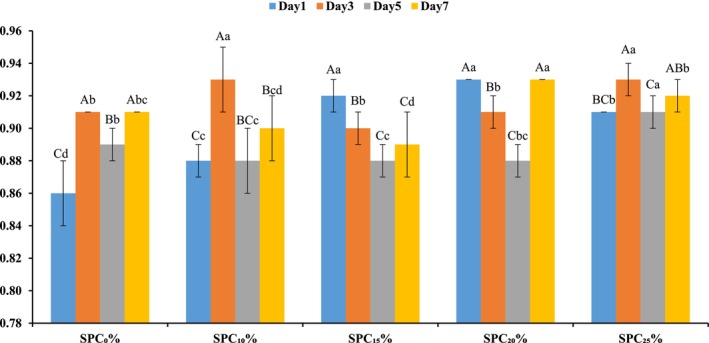
Effect of soy protein concentrate (SPC) supplementation on water activity of beef patties stored at refrigeration for 1–7 days. Different capital letters (A, B, C, D, E) within the same treatment indicate significant differences (*p* < 0.05) among storage days, while different lowercase letters (a, b, c, d, e) within the same storage day indicate significant differences (*p* < 0.05) among treatments (SPC levels). SPC_0%_ = Beef patties containing 0% soy protein concentrate (Control); SPC_10%_ = Beef patties containing 10% soy protein concentrate; SPC_15%_ = Beef patties containing 15% soy protein concentrate; SPC_20%_ = Beef patties containing 20% soy protein concentrate; SPC_25%_ = Beef patties containing 25% soy protein concentrate.

Findings for the water activity of SPC supplemented patties revealed a notable increase in its values from 0.86–0.93 on increasing the SPC supplementation level from 10% to 25% (Figure 1). Upon storage, on the 0th day the lowest water activity was recorded in control (SPC_0%_); whereas, on the 7th day, the highest results were recorded for SPC_15%_. Textured soy protein has lower fats, which allowed the protein to bind more freely with the water, so the water‐holding capacity increased (Hidayat et al. 2018). However, water release increases with higher soybean, leading to increased water activity in SPC‐treated patties. On the other hand, SPC_25%_ exhibited significantly higher a_w_ values than control on days 1, 3 and 5. Whereas, the a_w_ values on day 7 were significantly higher than those on day 1 for control and SPC_10%_ patties. During storage, the increased water activities may increase microbial growth (Velasco et al. 2014; Osei Tutu et al. 2019; Asiedu et al. 2025).

#### 3.1.3 Lipid Oxidation or TBARS

**FIGURE 2:**
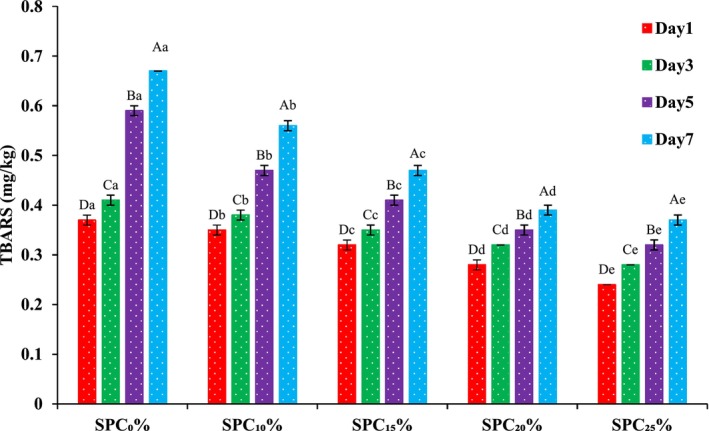
Effect of soy protein concentrate (SPC) supplementation on TBARS of beef patties stored at refrigeration for 1–7 days. Different capital letters (A, B, C, D, E) within the same treatment indicate significant differences (*p* < 0.05) among storage days, while different lowercase letters (a, b, c, d, e) within the same storage day indicate significant differences (*p* < 0.05) among treatments (SPC levels). SPC_0%_ = Beef patties containing 0% soy protein concentrate (Control); SPC_10%_ = Beef patties containing 10% soy protein concentrate; SPC_15%_ = Beef patties containing 15% soy protein concentrate; SPC_20%_ = Beef patties containing 20% soy protein concentrate; SPC_25%_ = Beef patties containing 25% soy protein concentrate.

The addition of SPC to beef patties caused a decrease in TBARS that was found to be significant (*p* < 0.05) slightly decreasing from 0.35% to 0.24% for SPC_10%_–SPC_25%_, respectively (Figure 2). However, control (T_0_) showed a significant increase (*p* < 0.05) in TBARS from 0.37% to 0.67% between 1 and 7 days of storage. The increase of SPC from 10% to 25% (SPC_10%_–SPC_25%_) and storage for 1–7 days resulted in a significant (*p* < 0.05) decline in TBARS of all treatments (SPC_10%_–SPC_25%_) when compared to the control (SPC_0%_). The SPC_25%_ sample achieved the lowest TBARS value from 0.24% to 0.37% during its storage period from 1 to 7 days. The findings of this study agree with a study conducted by Alakali et al. (2010) and Kumar and Sharma (2004) who reported that the addition of plant ingredients decreased TBARS values. Soy protein has a negligible amount of essential fats but higher concentrations of phenolics, which act as an antioxidant (Peña‐Ramos and Xiong 2003). Patties containing SPC had lower fat content which reduces the substrate for oxidation showed lower lipid oxidation compared to the control. Similar to the study by Ulu (2004), whey protein concentrate combined with SPC had lower lipid oxidation in meatballs. The lipid oxidation by‐products increasing during storage were similar to the findings of Liu et al. (2022). The significant increase in TBARS values of beef patties with increasing storage time is similar to the findings of Kilic et al. (2010) and Ahmad et al. (2010), who stored buffalo meat emulsion sausage supplemented with textured soy proteins showed no effect on TBARS values of the buffalo meat emulsion sausage. Another study, also showed a significant effect of soy protein addition in delaying the lipid oxidation process as well as in preventing the beef patties' deterioration during storage (Guerrero et al. 2015).

#### 3.1.4 Microbial Analysis

**FIGURE 3:**
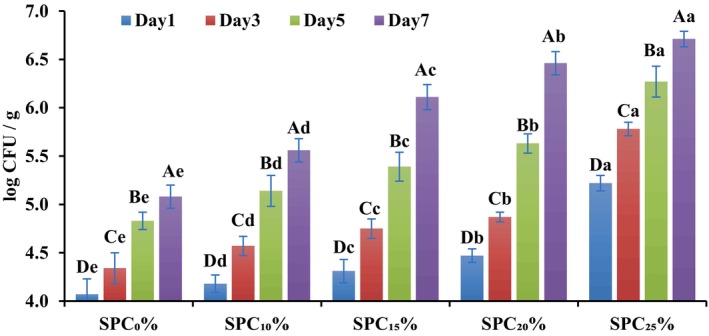
Effect of soy protein concentrate (SPC) supplementation on total plate count (TPC, log CFU/g) of beef patties stored at refrigeration for 1–7 days. Different capital letters (A, B, C, D, E) within the same treatment indicate significant differences (*p* < 0.05) among storage days, while different lowercase letters (a, b, c, d, e) within the same storage day indicate significant differences (*p* < 0.05) among treatments (SPC levels). SPC_0%_ = Beef patties containing 0% soy protein concentrate (Control); SPC_10%_ = Beef patties containing 10% soy protein concentrate; SPC_15%_ = Beef patties containing 15% soy protein concentrate; SPC_20%_ = Beef patties containing 20% soy protein concentrate; SPC_25%_ = Beef patties containing 25% soy protein concentrate.

The research findings indicated that the plate count of beef patties with soy proteins non‐significantly grew (*p* < 0.05) at 1–7 days from 4.18–5.22 for SPC_10%_–SPC_25%_, respectively (Figure 3). Similarly, control had a non‐significant (*p* < 0.05) increase from 4.07 to 5.08 in the total plate counts during the storage days 1–7. When the SPC ranged from 10% to 25% (SPC_10%_–SPC_25%_) and storage time was 1–7 days, there was a substantial (*p* < 0.05) increase in total plate counts of all beef patties. Out of all treatments tested, SPC_25%_ had the highest level of total plate counts (5.22) on the first day, compared to just 4.07 for the control sample. Product handling during processing is the cause of bacterial entry in the product (El‐Refai et al. 2014). The significant increase in bacterial count in this research is aligned with the results of Alakali et al. (2010) and Igyor et al. (2008), who supplemented Bambara groundnut flour in beef patties, and included melon kernel flour in beef sausages, respectively. The microbial count could be increased due to higher pH and water activity with increasing SPC concentration, as reported in Tables 1 and 2 and Figure 2. Similarly, the significantly higher log CFU/mL found in this study is in line with the findings of Noumo et al. (2021) with increasing storage period.

**TABLE 2:** Effect of soy protein concentrate (SPC) supplementation on weight loss, cook loss, cooking and cooking yield of beef patties stored under refrigeration at 1–7 days.

Days/treatments	SPC_0%_	SPC_10%_	SPC_15%_	SPC_20%_	SPC_25%_
Weight loss (%)					
1	0 ± 0^Ba^	0 ± 0^Ba^	0 ± 0^Ba^	0 ± 0^Aa^	0 ± 0^Ba^
3	0.08 ± 0.29^ABa^	0.08 ± 0.28^ABa^	0.17 ± 0.39^Ba^	0.08 ± 0.29^Aa^	0.08 ± 0.29^Ba^
5	0.33 ± 0.49^Aa^	0.33 ± 0.49^Aa^	0.25 ± 0.45^ABa^	0.08 ± 0.29^Aa^	0.25 ± 0.45^ABa^
7	0.25 ± 0.45^ABa^	0.25 ± 0.45^ABa^	0.5 ± 0.52^Aa^	0.17 ± 0.39^Aa^	0.41 ± 0.51^Aa^
Cook loss (%)					
1	29.58 ± 1.15^Ba^	27.89 ± 1.75^Cb^	26.74 ± 1.19^Cb^	21.39 ± 1.75^Cc^	16.93 ± 1.63^Dd^
3	28.87 ± 1.59^Ba^	27.72 ± 1.63^Ca^	29.3 ± 1.08^Bab^	28.35 ± 2.32^Ba^	25.82 ± 1.7^Bb^
5	33.5 ± 2.18^Aa^	31.04 ± 0.9^Bb^	32.33 ± 1.06^Aab^	30.98 ± 1.6^Ab^	23.79 ± 1.48^Cc^
7	34.63 ± 1.05^Aa^	32.99 ± 1.52^Ab^	33.01 ± 0.51^Ab^	31.91 ± 1.27^Ac^	28.8 ± 0.85^Ad^
Cooking yield (%)					
1	70.42 ± 1.15^Ad^	72.11 ± 1.75^Ac^	73.26 ± 1.19^Ac^	78.61 ± 1.75^Ab^	83.07 ± 1.63^Aa^
3	71.13 ± 1.59^Ab^	72.28 ± 1.63^Ab^	70.7 ± 1.08^Bb^	71.65 ± 2.32^Bb^	74.18 ± 1.7^Ca^
5	66.5 ± 2.18^Bc^	68.96 ± 0.9^Bb^	67.67 ± 1.06^Cbc^	69.02 ± 1.6^Cb^	76.21 ± 1.48^Da^
7	65.37 ± 1.05^Bd^	67.01 ± 1.52^Cc^	66.99 ± 0.51^Cc^	68.09 ± 1.27^Cb^	71.2 ± 0.85^Ba^

*Note:* Capital lettering exhibiting statistically significant (*p* < 0.05) mean values in a column, while; small lettering exhibiting statistically significant (*p* < 0.05) mean values in a row. SPC_0%_ = 0% soy protein concentrate supplemented beef patties (Control), SPC_10%_ = 10% soy protein concentrate supplemented beef patties, SPC_15%_ = 15% soy protein concentrate supplemented beef patties, SPC_20%_ = 20% soy protein concentrate supplemented beef patties, SPC_25%_ = 25% soy protein concentrate supplemented beef patties.

### 3.2 Assessment for Commercial Suitability and Industrial Productivity

#### 3.2.1 Weight Loss During Storage, Cooking Loss (%) and Cooking Yield (%)

Results from Table 2 showed that SPC addition only resulted in less than 0.5% losses during the refrigerated storage for 7 days at 4°C. Moreover, the replacement of beef with SPC in beef patties significantly decreased cook loss % compared to control samples with increasing concentration from 10% to 25% over the entire storage period at 4°C, while at day 5 and day 7 exhibited significantly higher cook loss % compared to day 1 for all the SPC‐enriched beef patties. The reverse was true for cook yield %. The ability of meat to retain water before and after cooking is of prime concern for the processors, as it controls product yield and other quality attributes. The lower CL% in SPC‐added beef patties could also be attributed to higher WHC (Hale et al. 2002), mainly because of the rehydration and gelling properties of SPC (Bakhsh et al. 2021; Mensah et al. 2026).

#### 3.2.2 Diameter Shrinkage (Effects on Water, Texture, Sensory)

The results for the diameter shrinkage (Table 4) indicated that increasing SPC concentration significantly (*p* < 0.05) decreased the shrinkage (%) of patties at 4°C compared to control samples, with the lowest shrinkage for SPC_25%_ at all the storage intervals that is 19% at day 1, and 22.25% at day 7. Moreover, increasing storage duration resulted in significant expansion of Control, SPC 15% and SPC 25% beef patties. The increasing concentration of SPC contributed to the decrease in shrinkage due to protein denaturation (Gujral et al. 2002). Moreover, greater interaction of plant‐origin proteins with fats in the beef patties could also be attributed to shrinkage (Serdaroglu 2006; Acheampong et al. 2024). However, the increasing storage period contributed to the expansion of patties possibly due to the moisture absorption capability of plant materials in the patties (Wang et al. 2023). Moreover, the reason for expansion during storage could be the ability of SPC to hinder the denaturation of connective tissue proteins during the refrigerated storage at 4°C (Bakhsh et al. 2021). Further, the texture profile of soy protein‐based beef patties resulted in a non‐significant change in textural attributes up to 14 days of storage; however, deterioration was observed in control samples only (Guerrero et al. 2015). Similarly, another study by Ball et al. (2021) improved the sensor features of beef patties as a result of adding the plant proteins in beef patties which could be linked with the underlying mechanism of water absorption, and hygroscopicity of plant proteins and the ability to bind moisture.

#### 3.2.3 Expressible Moisture and Water Holding Capacity

Increasing the concentration of SPC significantly (*p* < 0.05) increased the water holding capacity (WHC, %) values compared to control samples at all the storage intervals (Table 4). In contrast, the increasing number of storage days at the refrigerated temperature resulted in significant loss of WHC % of SPC‐added beef patties as compared to day 1. Our findings were consistent with the study of Das, Anjaneyulu, Gadekar, et al. (2008) and Lee et al. (2021) who reported that the WHC in meat products increased by adding soy protein. The increase in WHC in this study could be due to an increase in pH in this study (Table 1), which was attributed to the total negative charge of myofibrillar proteins increasing (Feng et al. 2013; Lee and Chin 2016; Souissi et al. 2016) who reported the enhancement of WHC in sausages substituted with plant ingredients. The higher levels of SPC in beef patties contributed to higher WHC % due to the presence of higher water‐soluble proteins in SPC in this study. Moreover, the low isoelectric point of soy proteins allowed the patties in this study to retain more water as compared to the control (Hidayat et al. 2018). In addition, the proteins faced lesser hindrance from inherently lower fat content in SPC, thereby exhibiting greater affection towards free water in the SPC‐incorporated beef patties. Moreover, the insoluble polysaccharide dietary fiber in SPC could also contribute to water‐polysaccharide interactions in the patties (Asgar et al. 2010). However, the loss of WHC during storage could be attributed to the oxidative conditions over storage that disrupt the order and integrity of muscle cells and limit the ability of myogenic fibers to absorb water, thereby reducing the WHC of the patties Bao and Ertbjerg (2019).

### 3.3 Consumer Acceptability

#### 3.3.1 Instrumental Color

The effect of beef substitution with varying levels of SPC on CIE L*, a* and b* color coordinates of beef patties was shown in Table 3. The lightness (L*) values indicated the perceived brightness of the beef patties, through estimation of visual attributes and potential consumer perception. Table 3 indicated that the addition of SPC (SPC 20% and 25%) contributed to increased L* values of the raw beef patties on all the storage days at 4°C; 15% showed higher values on days 1, 3, and 7, while 10% showed higher values on day 3 and day 7, than the control group (SPC_0%_). The redness (a*) values of meat are considered an indicator of meat freshness. Soy protein binds more water and causes more light reflection, contributing to the higher L* values in SPC ‐added patties than in the control (Wong et al. 2019). In addition, the a* values of the SPC_10%_ supplemented patties were significantly higher than control (SPC_0%_) on all storage days. Moreover, the a* values of the 15%–25% replacement of beef with SPC in the beef patties significantly decreased with increasing SPC concentration on day 3, day 5 and day 7. The results of this study were in contrast to the findings of Lee et al. (2021), who reported a decrease in L* values using plant proteins. The increase in yellowness (b*) values was due to the color of the SPC, which was yellowish‐brown. Moreover, b* values of patties having 15%–25% SPC were significantly higher than control (SPC_0%_) on all the storage days at 4°C, while those having 10% SPC were significantly higher than control on day 1 and day 7 only. SPC addition lowered myoglobin content and caused lower a* values due to the yellowish color imparted by SPC (Asgar et al. 2010; Acheampong et al. 2024; Osei Tutu et al. 2024a). The increase in b* values in this study was similar to the outcomes of Savadkoohi et al. (2014), who reported that plant fibers and protein addition significantly increased b* values. Our results are similar to the findings of Hidayat et al. (2018), in which the addition of SPC in sausages increased L* and b* values, but in contrast to the findings of Akesowan (2010) reported a negative correlation (increasing b* and decreasing L*) in soy protein‐modified pork patties. The effect of refrigerated storage was variable on the CIE L*, a*, b* coordinates of SPC‐added beef patties. On the other hand, day 7 storage at 4°C did not result in the loss of L* in SPC_25%_ samples, but there was significant loss in SPC_0%_–SPC_20%_. Both day 3 and day 5 storage intervals resulted in the loss of L* in SPC 10%–20%. a* values of all the SPC‐supplemented samples were notably lesser than control on all the storage days at 4°C. Myoglobin and hemoglobin cause redness in meat (Zhang et al. 2013), and with the passage of storage days, deterioration of these meat proteins due to lipid oxidation decreased redness values.

**TABLE 3:** Effect of soy protein concentrate (SPC) supplementation on chromatic prolife of beef patties stored under refrigeration at 1–7 days.

Days/treatments	SPC_0%_	SPC_10%_	SPC_15%_	SPC_20%_	SPC_25%_
L*					
1	42.97 ± 0.82^Ad^	43.56 ± 0.31^Acd^	44.23 ± 0.53^Abc^	45.01 ± 0.6^Aab^	45.52 ± 2.43^Aa^
3	42.37 ± 0.58^Ad^	43.25 ± 0.36^Bc^	43.66 ± 0.48^Bbc^	44.04 ± 0.79^Bb^	45.25 ± 0.43 ^Aa^
5	42.23 ± 0.75^Ac^	42.54 ± 0.36^Cbc^	42.56 ± 0.4^Cbc^	42.86 ± 0.48^Cb^	44.69 ± 0.38 ^Aa^
7	39.2 ± 2.1^Bc^	42.29 ± 0.17^Cb^	42.79 ± 0.36^Cb^	42.92 ± 0.41^Cb^	44.75 ± 0.41 ^Aa^
a*					
1	18.12 ± 0.41^Ab^	19.05 ± 0.38^Aa^	18.32 ± 0.65^Ab^	17.36 ± 0.28^Ac^	17.29 ± 1.17^Ac^
3	14.66 ± 0.18^Bbc^	15 ± 0.34^Ba^	14.8 ± 0.31^Bab^	14.49 ± 0.35^Bcd^	14.23 ± 0.39^Bd^
5	12.66 ± 0.26^Ce^	15.07 ± 0.16^Ba^	14.47 ± 0.26^Bb^	13.69 ± 0.33^Cc^	12.97 ± 0.34^Cd^
7	12.74 ± 1.13^Cc^	14.14 ± 0.34^Ca^	13.95 ± 0.29^Cab^	13.33 ± 0.26^Db^	12.37 ± 0.32^Cc^
b*					
1	10.28 ± 0.35^Ab^	11.31 ± 0.34^Aa^	11.32 ± 0.35^Aa^	11.4 ± 0.34^Aa^	11.92 ± 1.3^Aa^
3	9.11 ± 0.23^Bc^	9.17 ± 0.47^Bc^	10.3 ± 0.22^Bb^	10.68 ± 0.43^Bab^	11.16 ± 0.42^Aa^
5	8.11 ± 0.06^Cc^	7.46 ± 0.3^Cd^	8.76 ± 0.21^Cb^	8.89 ± 0.11^Cb^	9.74 ± 0.33^Ba^
7	5.74 ± 0.72^Dd^	6.25 ± 0.14^Dc^	7.64 ± 0.13^Db^	7.81 ± 0.13^Dab^	8.18 ± 0.21^Ca^

*Note:* Capital lettering exhibiting statistically significant (*p* < 0.05) mean values in a column, while; small lettering exhibiting statistically significant (*p* < 0.05) mean values in a row. SPC_0%_ = 0% soy protein concentrate supplemented beef patties (Control), SPC_10%_ = 10% soy protein concentrate supplemented beef patties, SPC_15%_ = 15% soy protein concentrate supplemented beef patties, SPC_20%_ = 20% soy protein concentrate supplemented beef patties, SPC_25%_ = 25% soy protein concentrate supplemented beef patties.

#### 3.3.2 Texture Profile Analysis

Only SPC_20%_ and SPC_25%_ exhibited an increase in hardness of the patties, while the rest of the samples did not exhibit statistical differences from the control, as well as with the increasing storage intervals. However, on day 5, the SPC samples were less gummy than the controls (Table 4). There was no statistically significant difference found between mean gumminess and cohesiveness values among all treatments (*p* ≥ 0.05). The results of this study were in contrast to the findings of Shen et al. (2022) and Akesowan (2010), who reported the decrease in hardness by adding pea proteins to beef patties, and decreased hardness of pork patties due to rehydrated soy protein, respectively. The higher capability of rehydrated soy protein to retain moisture in the patties could contribute to a reduction in the hardness of SPC supplemented patties, while the ripening of plant protein added to the meat blend due to lipid oxidation could increase meat hardness (Ahmad et al. 2010). In addition, soy protein could fill the interstitial spaces with water within the meat batter protein matrix (Lee et al. 2021). The textural attributes did not change significantly during storage. However, the excessive hardness of SPC_25%_ could be attributed to higher fibers in SPC itself. Moreover, the probable reason for the increased hardness is the tougher network formation due to the denaturation of myofibrillar proteins in beef, which increases compression resistance (Kamani et al. 2019). Also, a study by Guerrero et al. (2015) elucidated the maintenance of the texture of soy protein‐beef patties on completion of the storage duration for 14 days.

**TABLE 4:** Effect of soy protein concentrate (SPC) supplementation on texture and techno‐functional attributes of beef patties stored under refrigeration at 1–7 days.

Days/treatments	SPC_0%_	SPC_10%_	SPC_15%_	SPC_20%_	SPC_25%_
Hardness (*N*)					
1	28.96 ± 8.01^Ba^	28.06 ± 0.94^Ba^	26.5 ± 3.86^Aa^	26.23 ± 4.57^Ba^	25.19 ± 4.24^Ba^
3	32.18 ± 1.14A^Ba^	32.3 ± 9.4^Aba^	30.93 ± 4.7^Aab^	31.44 ± 1.99^Aab^	26.36 ± 3.97^ABb^
5	34.92 ± 4.48^Aab^	37.74 ± 5.39^Aa^	31.6 ± 2.24^Aab^	34.73 ± 1.03^Abc^	29.3 ± 2.93^ABc^
7	33.55 ± 5.22^ABa^	30.14 ± 1.76^Ba^	32.62 ± 9.21^Aa^	31.47 ± 4.33^Aa^	29.89 ± 3.92^Aa^
Cohesiveness (*N*)					
1	0.59 ± 0.05^Aa^	0.59 ± 0.02^Aa^	0.61 ± 0.05^Aa^	0.6 ± 0.05^Aa^	0.61 ± 0.03^Aa^
3	0.57 ± 0.03^Aa^	0.59 ± 0.11^Aa^	0.57 ± 0.08^Aa^	0.57 ± 0.06^Aa^	0.59 ± 0.05^Aa^
5	0.55 ± 0.11^Aa^	0.57 ± 0.1^Aa^	0.57 ± 0.03^Aa^	0.56 ± 0.1^Aa^	0.56 ± 0.03^Aa^
7	0.54 ± 0.07^Aa^	0.55 ± 0.04^Aa^	0.62 ± 0.15^Aa^	0.59 ± 0.07^Aa^	0.6 ± 0.09^Aa^
Gumminess (*N*, mm)					
1	17.55 ± 6.48^Aa^	16.67 ± 0.58^Aa^	16.15 ± 3.34^Ba^	15.99 ± 3.82^Ba^	15.37 ± 3.25^Aa^
3	18.37 ± 1.03^Aab^	18.72 ± 4.72^Aa^	17.31 ± 1.22^ABab^	17.85 ± 1.9^ABab^	15.55 ± 3.28^Ab^
5	19.37 ± 5.48^Ab^	19.78 ± 3.65^Aa^	18.1 ± 0.52^ABa^	19.28 ± 3.37^Aa^	16.37 ± 1.06^Aa^
7	17.98 ± 2.55^Aab^	16.47 ± 0.9^Ab^	19.14 ± 1.09^Aa^	18.47 ± 2.22^ABa^	17.67 ± 1.02^Aab^
Water holding capacity (%)					
1	76.44 ± 1.05^Ab^	76.18 ± 0.54^Ab^	80.33 ± 1.5^Aa^	80.49 ± 0.81^Aa^	80.71 ± 0.62^Aa^
3	70.75 ± 0.56^Cd^	73.98 ± 1.06^Bc^	76.4 ± 1.15^Bb^	77.5 ± 0.63^Ba^	76.53 ± 0.5^Cb^
5	71.88 ± 0.47^Bd^	73.23 ± 0.41^Bc^	75.04 ± 1.29^Bb^	72.9 ± 0.48^Dc^	78.59 ± 1^Ba^
7	68.79 ± 0.97^Dc^	72.11 ± 1.8^Cb^	72.55 ± 4.43^Cb^	76.05 ± 0.57^Ca^	73.85 ± 0.59^Db^
Shrinkage (%)					
1	23.98 ± 1.23^Ba^	22.35 ± 1.09^Bb^	22.01 ± 1^Bb^	21.75 ± 0.41^Ab^	19.02 ± 1.37^Bc^
3	27.03 ± 0.98^Aa^	23.99 ± 1.22^Ab^	23.48 ± 0.44^ABbc^	22.52 ± 1.25^Acd^	21.35 ± 1.42^Ad^
5	26.69 ± 0.95^Aa^	23.01 ± 0.82^ABc^	24.66 ± 1.34^Ab^	23.04 ± 1.84^Ac^	22.5 ± 0.94^Ac^
7	25.68 ± 1.72^Aa^	22.98 ± 1.12^ABbc^	24.38 ± 2.14^Aab^	23.01 ± 0.8^Abc^	22.25 ± 1.32^Ac^

*Note:* Capital lettering exhibiting statistically significant (*p* < 0.05) mean values in a column, while; small lettering exhibiting statistically significant (*p* < 0.05) mean values in a row. SPC_0%_ = 0% soy protein concentrate supplemented beef patties (Control), SPC_10%_ = 10% soy protein concentrate supplemented beef patties, SPC_15%_ = 15% soy protein concentrate supplemented beef patties, SPC_20%_ = 20% soy protein concentrate supplemented beef patties, SPC_25%_ = 25% soy protein concentrate supplemented beef patties.

#### 3.3.3 Sensory Analysis

**FIGURE 4:**
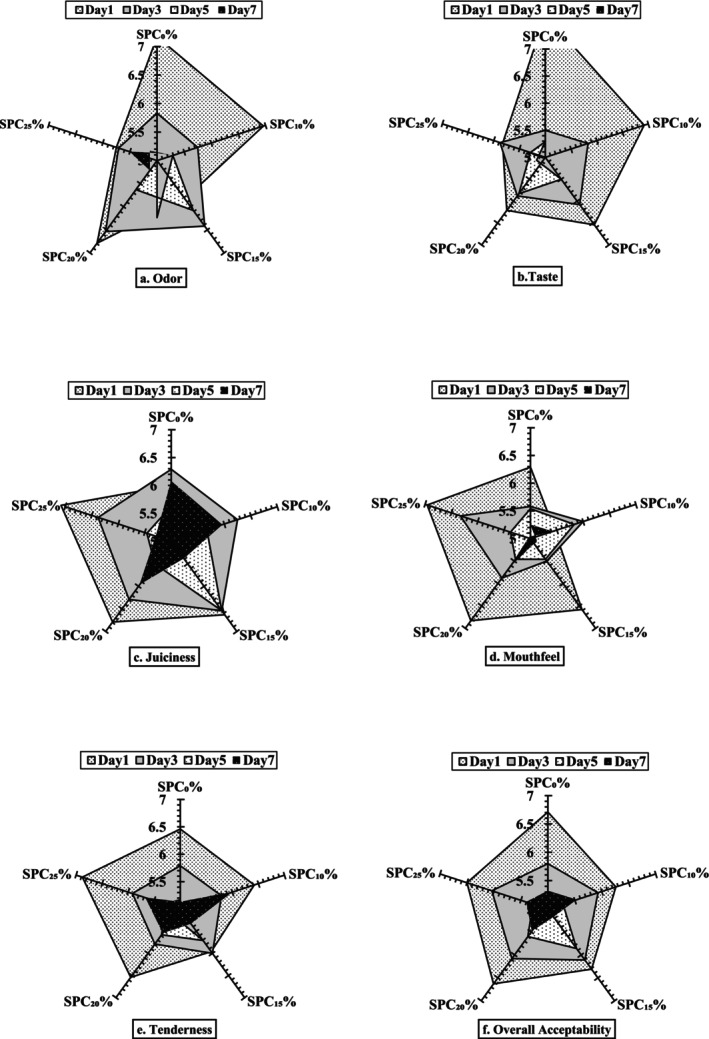
Effect of soy protein concentrate (SPC) supplementation on the sensory attributes of beef patties stored at refrigeration for 1–7 days. SPC_0%_ = Beef patties containing 0% soy protein concentrate (Control); SPC_10%_ = Beef patties containing 10% soy protein concentrate; SPC_15%_ = Beef patties containing 15% soy protein concentrate; SPC_20%_ = Beef patties containing 20% soy protein concentrate; SPC_25%_ = Beef patties containing 25% soy protein concentrate.

Kilic et al. (2010) reported a decreased meat flavor perception intensity by panelists of texturized soy protein added meatballs. The sensory taste score significantly (*p* < 0.05) decreased as compared to the control samples only on day 1; however, although the taste score also decreased with increasing storage intervals, the substitution with higher SPC concentrations withstood taste alteration for a longer time (Figure 4). Addition of SPC resulted in enhanced odor compared to control samples which could be linked to the presence of aromatic volatile compounds in SPC itself. The alterations of odor and taste of SPC patties in this study could be attributed to the formation of various volatile compounds (Kasaiyan et al. 2023). The sensory juiciness score significantly decreased for SPC_15%_–SPC_25%_ in comparison to the control samples only on day 1; however, the higher the concentration of SPC in the patties the more was the reduction in juiciness with the storage interval. These findings are similar to the results of Serdaroglu (2006), who reported increased juiciness with the addition of plant sources of oats in beef patties. The decrease in juiciness could be related to the higher fiber content of SPC‐substituted patties (Lee et al. 2021; Hong et al. 2022). The sensory mouthfeel, tenderness, and overall acceptability scores of SPC‐substituted patties were not affected by the impact of concentration; however, storage intervals decreased their scores. According to Odiase et al. (2013), the 25% soy flour inclusion can be incorporated into meat products without affecting consumer preferences but 15% were liked very much. Over the refrigerated storage period, sensory scores for different attributes decreased but remained within acceptable limits till the 7th day of the storage (Ahmad et al. 2010). The decreasing trend in all sensory scores was due to the production of off‐flavor volatile compounds because of lipid oxidation over time (Asgar et al. 2010; Asiedu et al. 2025, 2026; Obeng et al. 2025). Similarly, another study by Guerrero et al. (2015) exhibited preservation of sensorial attributes of beef patties prepared with the addition of soy proteins for 14 days which could be linked to the anti‐staling properties of proteins.

## 4 Conclusion

SPC addition in beef patties improved textural, sensorial attributes and microbial stability. Supplementation of SPC_10%_ in beef patties yielded significantly lower cooking losses while, reducing shrinkage, and increasing the cooking yields. Higher concentrations of SPC resulted in less off‐flavors during storage indicating anti‐microbial and anti‐oxidative and lipid‐oxidation protective effects. Beef patties prepared with the addition of SPC_25%_ portrayed better tenderness, juiciness and overall acceptability for consumers. SPC shows potential as a natural substitute for animal proteins in processed meat products since it is cost effective, sustainable and accessible while expanding protein choice. In the future, further studies may be conducted to effectively approach new processing methods and interactions of ingredients to maximize the functionality of SPC in next‐generation meat products.

## Author Contributions

**Muhammad Moazzam:** data curation, investigation, methodology. **Sher Ali:** supervision, conceptualization, writing original draft. **Muhammad Ammar Khan:** supervision, conceptualization, methodology. **Muhammad Waseem:** software, review and editing, R&D, and statistics. **Muhammad Rizwan Javed** and **Nasir Rajput:** endnote referencing, statistics. **Tawfiq Alsulami:** writing review editing, tables, and figures. **Kashif Nauman:** writing review editing, methodology and software. **Crossby Osei Tutu:** formal analysis, methodology, software, validation, visualization, writing review editing, resources.

## Conflicts of Interest

The authors declare no conflicts of interest.

## Data Availability

The data for the study is incorporated in the article or referenced within the article.
